# Simultaneous Determination of Pigments, Tocopherols, and Squalene in Greek Olive Oils: A Study of the Influence of Cultivation and Oil-Production Parameters

**DOI:** 10.3390/foods9010031

**Published:** 2019-12-29

**Authors:** Ioannis Martakos, Marios Kostakis, Marilena Dasenaki, Michalis Pentogennis, Nikolaos Thomaidis

**Affiliations:** Laboratory of Analytical Chemistry, Department of Chemistry, National and Kapodistrian, University of Athens, Panepistimiopolis Zographou, Athens 15771, Greece; johnmrtk@chem.uoa.gr (I.M.); makostak@chem.uoa.gr (M.K.); pentogmi@otenet.gr (M.P.); ntho@chem.uoa.gr (N.T.)

**Keywords:** olive oil, high-performance liquid chromatography, pigments, tocopherols, squalene, cultivation parameters, olive oil-production parameters

## Abstract

A new facile and fast method was developed in this study for the determination of pigments (chlorophylls and carotenoids), tocopherols (α-, sum of (β + γ), and δ), and squalene in olive oil. This method consisted of a dilution of olive oil in 2-propanol, followed by reversed phase-high-pressure liquid chromatography equipped with a diode array detector (RP-HPLC-DAD). Chromatographic separation was performed using a C18 column, while the mobile phase consisted of acetonitrile and methanol using a gradient elution program. The methodology was optimized, validated, and applied to the analysis of 452 samples of Extra Virgin Olive Oil (EVOOs) and Virgin Olive Oil (VOOs) originated from five islands of the Northeastern Aegean Region, in Greece. From the obtained results, it was indicated that the carotenoid, tocopherol, and squalene content was relatively high, while the chlorophyll content was low. Furthermore, the acquired results were studied and compared in order to obtain useful information about the correlation of the concentration levels of these compounds in olive oil to different cultivation and olive oil production parameters. Several parameters were found to play a significant role on the pigment and antioxidant content of olive oil, such as the olive tree variety, geographical origin, fruit maturation stage during harvesting, and addition of water during malaxation, while other parameters such as the altitude of cultivation, the type of farming (organic or conventional), and the type of olive mill did not seem to affect the levels of these compounds.

## 1. Introduction

Olive oil is a significant and very beneficial ingredient of the Mediterranean diet. Extra Virgin Olive Oil is obtained from the olive fruits of *Οlea Europaea L*. trees, by mechanical extraction processes under controlled temperature conditions, which result in the preservation of its quality characteristics and bioactive compounds [1]. Olive oil has been produced since ancient years and has always been a very healthy and nutritious component of the human diet, mainly because of the very rich and pure bioactive content. 

Compounds present in olive oil have been studied thoroughly in a great number of studies in the past years. From the non-saponifiable class of olive oil, pigments, tocopherols, and hydrocarbon fraction have gained great attention due to their antioxidant activity, mainly by reacting with free radicals resulting in the preservation of other important compounds. Pigments are divided into two important groups, carotenoids and chlorophylls. Both pigment groups are exclusively synthesized by plants and their derivatives, thus the only way of infiltration to the human body is through a balanced diet. Olive oil carotenoids (β-carotene, lutein, zeaxanthin, violaxanthin, and other minor xanthophylls in minor percentages) are natural antioxidants and have sufficient and beneficial properties for human health. Lutein protects the eye retina from oxidative damage, while beta-carotene appears to lower the risk of heart diseases [2,3,4]. Chlorophylls (chlorophyll a and a’, pheophytins a and a’, and other chlorophylls in minor percentages) are responsible for the green-yellowish color of olive oil. They are used as indicators to assess the quality and inveteracy of olive oil. Low concentrations of pheophytins and pyropheophytin indicate a fresh, well-stored olive oil, while high concentrations of these compounds indicate an old, poorly preserved olive oil [5,6]. 

Tocopherols (α, β, γ, and δ) are natural, lipid-soluble antioxidants. They belong to the Vitamin E group and are present in most plant and vegetable oils. They contribute to the oxidative stability of olive oil, as stated in a number of studies [7,8]. Squalene represents more than 90% of the hydrocarbons in olive oil. Many scientific articles have been published in order to study the health benefits of squalene and its potential anti-cancer capabilities [9,10].

The concentrations of pigments, tocopherols, and squalene in olive oil tend to differ depending on the geographical origin, the variety of the olive fruit, the cultivation parameters, the fruit maturation stage during ripening, and the oil production parameters (oil press system, centrifugation system, addition of water during malaxation of olive paste, malaxation time, malaxation temperature) [11,12,13,14,15]. In the literature, many studies have reported the connection of the quality of olive oil with the concentration of these compounds (pigments and antioxidants), thus it is of great value to be able to determine the levels of these compounds with a fast, cheap, and effective methodology. In the majority of the published studies, high-performance liquid chromatography equipped with a diode array detector (HPLC-DAD) is used. This technique offers excellent separation and relatively low detection and quantification limits, while the detector provides very good signal stability. The diode array detector’s huge advantage is the simultaneous monitoring of pre-set wavelengths during the whole chromatogram time. Although there is a great number of studies that focus on the determination of these compounds in olive oil, to the best of our knowledge, none of them refers to a method for the simultaneous determination of these compounds in a single run. 

According to the IOC (International Olive Council), Greece is the third country in olive oil production worldwide, the leading country in olive oil consumption per capita per year, and the leading country in olive oil export [16]. The three primary olive tree cultivation regions in Greece are Crete, Peloponnese, and Lesvos Island. To the best of our knowledge so far, no comprehensive studies have been reported regarding the assessment of pigments, tocopherols, and squalene in Greek olive oils and the influence of cultivation and oil-production parameters to the concentration of these compounds in olive oil.

The aim of this study was the development and validation of a new, cost-effective, fast, and robust method for the simultaneous determination of pigments, tocopherols, and squalene in olive oil by HPLC-DAD. Furthermore, the method was applied to samples of extra virgin olive oil (EVOOs) and virgin olive oil (VOOs) of five Greek islands of the Northeastern Aegean Region of Greece, in order to obtain useful information about the chemical composition of Greek olive oils and their correlation with several parameters of cultivation and oil-production.

## 2. Materials and Methods

### 2.1. Chemicals and Reagents

Methanol (MeOH), ethanol (EtOH), and acetonitrile (ACN), HPLC grade, and diethyl ether and petroleum ether, analytical grade, were purchased from Fischer Scientific (Geel, Belgium). 2-propanol and acetone, HPLC grade, were purchased from Honeywell (Offenbach, Germany). All the standard compounds (lutein, chlorophyll A 87%, β-carotene, α-tocopherol, γ-tocopherol, δ-tocopherol, and squalene) were purchased from Sigma Aldrich (Stenheim, Germany). Stock solutions containing 1000 mg/L of each analyte were prepared in methanol for lutein, in ethanol for β-carotene and tocopherols, in diethyl ether for squalene, and in acetone for chlorophyll, and stored at −20 °C during the study. Intermediate standard solutions of 100–500 mg/L of each compound were prepared in 2-propanol.

Mixed working standard solutions were prepared from the intermediate solutions, in order to construct the calibration curves of the analytes used for quantification. For the optimization of the method, solid phase extraction (SPE) Strata Silica 1 g/6 mL cartridges (55 μm, 70 Å) were purchased from Phenomenex (California, USA). All samples were filtered using Chromafil Regenerated Cellulose (RC) syringe filters, 0.22 μm, purchased from Macherey-Nagel (Düren, Germany) prior to injection.

### 2.2. Samples

In total, 452 samples of EVOOs and VOOs (404 samples of EVOOs and 48 of VOOs) were acquired from five islands (Lesvos, Samos, Ikaria, Chios, and Fournoi) of the Northeastern Aegean Region, Greece. All samples were obtained in the 2017/2018 harvesting season and were divided into groups according to their different agronomical and oil-production parameters, such as olive fruit variety, altitude of cultivation, type of cultivation (conventional or biological), maturation stage during ripening, and also production in two-phase or three-phase mills and malaxation parameters (time, temperature, addition of water), among others. All information regarding the olive oil samples are presented in Table S1 in the Electronic Supplementary Material. 80% of the samples were obtained from the island of Lesvos, thus Lesvos was divided to seven geographical zones in order to facilitate the classification of the results regarding the corresponding factors (Supplementary Figure S1). 

### 2.3. Sample Preparation and Analysis

Samples were treated as follows: 100 μL of olive oil sample was weighted and dissolved in 900 μL 2-propanol. The mixture was filtered through a Chromafil RC 0.22 μm filter and an aliquot of 20 μL was injected into the HPLC system. The HPLC system used for the determination of the analytes was an Agilent 1200 series (Agilent, Santa Clara, CA, USA) equipped with an autosampler G1329A, degasser G1379B, column thermostat G1330B, binary pump G1312A, and diode array detector G1315D. The stationary phase was a reversed-phase Spherisorb ODS 2, 250 mm × 4.6 mm, i.d. 5 μm, purchased from Waters (Milford, MA, USA). The mobile phase consisted of (A) MeOH and (B) ACN, using the following gradient: 50% (A) for seven min (t = 0–7), then increased to 100% (A) in 5 min (until t = 12 min) and then stable in 100% (A) for three min (t = 12–15). After that, the % (A) is again reduced to 50% for another 12 min (t = 15–27 min). The flow rate was 1.0 mL/min and the overall analysis time was 27 min. DAD was set at 410 and 660 nm for chlorophylls, 430 and 450 nm for carotenoids, 295 nm for tocopherols, and 210 nm for squalene (Table 1). All compounds were identified through their retention times and spectral data and were quantified using standard calibration curves. Standard solutions for pheophytin and pyropheophytin were not available, thus they were not quantified. Pheophytin and pyropheophytin were tentatively identified with DAD spectra reported by Hornero-Mendez et al. [17]. As shown in Supplementary Figure S2, pheophytin and pyropheophytin present the exact same absorbance spectra, but pheophytin is eluted earlier than pyropheophytin (7–9 min). Β- and γ-tocopherols were quantified as a sum using γ-tocopherol standard, as also reported by Gliszczyńska-Świgło et al. [18]. The software used for the control of the HPLC system and the data treatment was the Agilent LC Chemstation Rev. B.01.03-SR2 (204). For the statistical evaluation of the results, analysis of variance (ANOVA) was performed using the Data Analysis tool of Microsoft Excel (Microsoft, WA, USA). In general, ANOVA is used to determine if there are any significant statistical differences between the means of unrelated groups of data. In this study, we used ANOVA to compare the results given by olive oils of different origin, different varieties, and different oil-producing parameters.

### 2.4. Method Validation

Regarding the method validation, the parameters evaluated were the linearity range, precision, recovery, and the methods limit of detection (LOD) and limit of quantification (LOQ). Due to the squalene’s increased concentration in olive oil, there was a risk of surpassing the linear range due to the self-absorption phenomenon. Thus, for the validation, we chose an old olive oil sample (from the harvest year 2016/2017) which we analyzed and verified that it contained a relatively low squalene concentration. All target analytes were determined in this reference olive oil sample and the concentrations were subtracted to calculate all validation parameters. 

Linearity was assessed by constructing calibration curves for each compound, using standard solutions prepared in six different concentrations. Precision was evaluated through repeatability and intermediate precision experiments and was expressed through %relative standard deviation (%RSD). For the repeatability, three replicates of the reference olive oil sample were spiked with the analytes at three different concentrations (β-carotene: 50–100–200 mg/kg, lutein 10–50–100 mg/kg, α-tocopherol 100–500–1000 mg/kg, γ-tocopherol 100–200-500 mg/kg, δ-tocopherol 100–500–1000 mg/kg, squalene 100–500–1000 mg/kg, chlorophyll 100–500–1000 mg/kg) and the samples were analyzed in six replicates under the same conditions (same analyst in one laboratory day). For the intermediate precision, three replicates of the reference olive oil sample were spiked with the analytes at the same concentrations and the samples were analyzed in six replicates during three different laboratory days. For the LOD and LOQ estimation, ten replicates of the reference olive oil sample were spiked with the analytes at low concentrations (β-carotene 1.00 mg/kg, lutein 0.26 mg/kg, α-tocopherol 20.0 mg/kg, γ-tocopherol 4.7 mg/kg, δ-tocopherol 23.0 mg/kg, squalene 11.8 mg/L, chlorophyll 4.0 mg/kg). The spiked sample was analyzed with the proposed methodology.

## 3. Results and Discussion

### 3.1. Method Development and Validation

During the development of the method, two different protocols were tested: one using solid phase extraction (SPE) and one simple dilution protocol. SPE was tested in order to pre-concentrate our samples, and as a further clean-up to minimize the background. For the SPE methodology, 1 g of oil sample was dissolved in n-hexane (2 mL). The sample was then loaded to the SPE Strata Silica 1 g/6 mL cartridges (55 μm, 70 Å). Diethyl ether and petroleum ether (1:9, 10 mL) were used in order to wash out the non-polar compounds and acetone (10 mL) was used as the eluent for the pigments. Spiked samples were analyzed to evaluate the efficiency of the SPE protocol and it was observed that the tocopherols and beta-carotene were washed out with the non-polar class of solvents. Furthermore, the recoveries of the spiked samples were <80% for squalene and chlorophyll. Thus, we developed a single-step dilution method for sample pretreatment, choosing 2-propanol as the dilution solvent [19].

**Figure 1:**
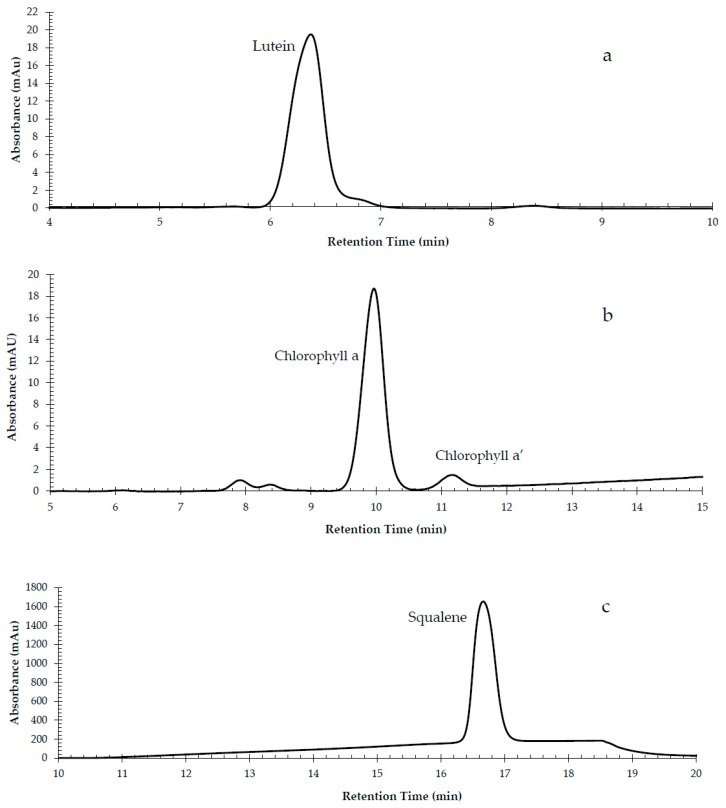

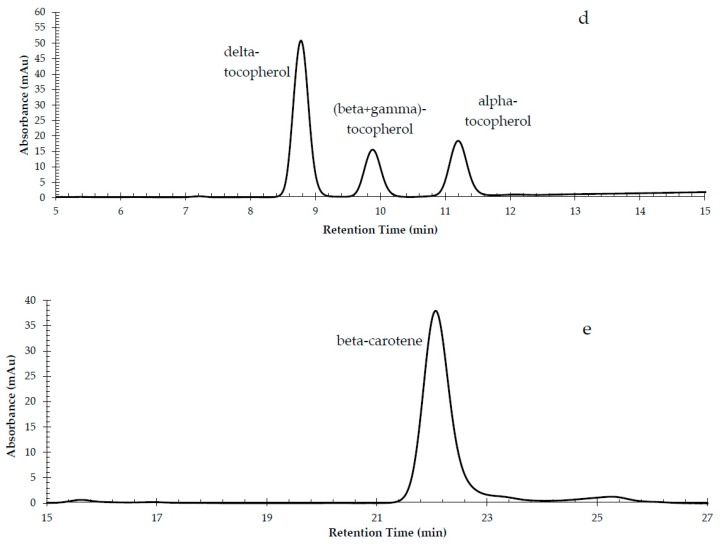
Chromatogram of a mixed standard solution of (**a**) lutein 1 mg/L, (**b**) chlorophyll a and a’ 10 mg/L, (**c**) squalene 250 mg/L, (**d**) δ-tocopherol 50 mg/L, (β + γ)-tocopherol 25 mg/L, α-tocopherol 25 mg/L, and (**e**) β-carotene 5 mg/L.

Regarding the chromatographic conditions, the mobile phase was the only parameter optimized. At first, an isocratic elusion was used consisting of MeOH:ACN—50:50. However, the analysis time surpassed 27 min, resulting in a long analysis time, while the peak symmetry factors were not ideal (Supplementary Table S2). To optimize the determination, a gradient elution program was tested, that resulted in more symmetrical peaks with better separation and reduced analysis time (Supplementary Table S2). That program is described in Section 2.3. The obtained chromatograms of all target compounds are presented in Figure 1.

The developed methodology was then validated showing high recoveries and excellent validation parameters. Regarding the linearity, as shown in Table 2, for all the compounds, the correlation coefficient was r^2^ > 0.99. For the evaluation of repeatability and intermediate precision, the %RSDs were calculated (Table 3 and Table 4), and the %recoveries were within the range of 80%–120% for all the samples. LODs and LOQs (Table 5) were estimated using Equations (1) and (2) in mg/L and mg/Kg, with mg/L referring to the final extract and mg/Kg referring to the initial olive oil sample, and were relatively low in comparison with the literature.

LOD = 3.3 × SD/b(1)

LOQ = 10 × SD/b(2)

where, SD = standard deviation of the low concentration sample, and b = slope of the calibration curve of each analyte.

### 3.2. Samples Results

All the samples in this study where produced during the 2017/2018 harvesting season and were analyzed during a time period of two months upon production. 

As expected, pheophytin A and pyropheophytin were only scarcely detected and they have not been quantified as a reference standard was not available. The absence of pheophytin A and pyropheophytin in the majority of the samples verified their freshness as well as their proper storage, since beside the inveteracy of olive oil, the concentration of these compounds depends on the storage temperature and conditions [20]. Chlorophyll a was detected in a small group of samples. As has been reported in previous papers, the amount of chlorophylls decreases when the maturation of the olive fruit increases [11]. The majority of the samples analyzed were produced using olive fruits of higher maturity level (black color).

In Table 6, lutein, beta-carotene, tocopherols, and squalene average concentrations are presented, along with the median concentration and standard deviation (SD). As presented, lutein is the main pigment compound detected in Northeastern Aegean olive oils, followed by beta-carotene, which was detected in about 65% of the samples. Regarding the tocopherols, the main compound is α-tocopherol, present in 100% of the samples, followed by (β + γ)-tocopherols. δ-tocopherol has not been detected to any of our samples. Squalene was also detected in high concentrations. All of the compounds’ average concentrations were similar to olive oil samples produced in Italy or Spain [1,15,21,22]. A great number of samples presented very high concentrations of carotenoids. However, about 6% of the samples contained β-carotene >2.5 mg/kg (17 samples), and 15% (67 samples) of the samples contained lutein >2.5 mg/kg while 30% (20 samples) contained lutein >3.5 mg/kg. 

Regarding the tocopherols, 6% (27 samples) of the samples contained α-tocopherol >250 mg/kg. The (β + γ) tocopherol content was very high in comparison with the literature [23,24,25]. Specifically, 33% of the samples (135 samples) contained (β + γ) tocopherols >25 mg/kg, while 34% (45 samples) of them contained >35 mg/kg. Finally, squalene was detected in high concentrations, with 15% (68 samples) of the samples containing great amounts of squalene (>3000 mg/kg). 

All these compounds, being detected in high concentrations in these EVOOs and VOOs, are indicators of the high quality and the high nutritional value of olive oils produced in the Northeastern Aegean Region.

### 3.3. Effect of Cultivation and Olive-Producing Parameters

**Figure 2:**
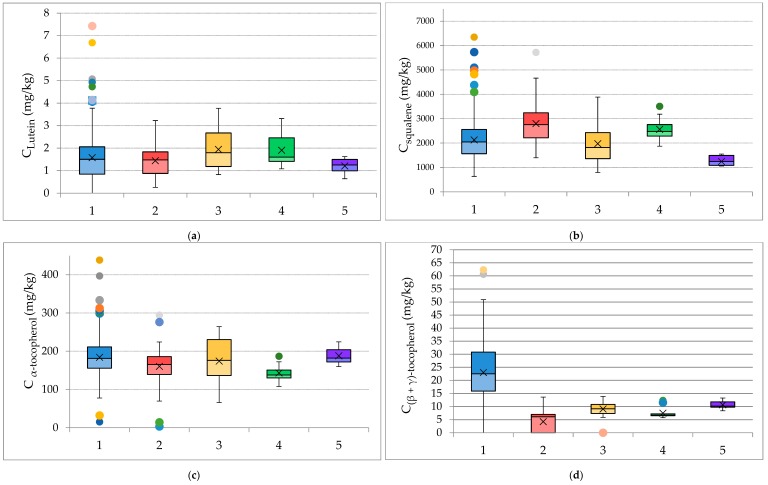
Box and whisker plots, presenting the concentrations of (**a**) lutein, (**b**) squalene, (**c**) α-tocopherol, and (**d**) (β + γ)-tocopherol according to the geographical origin. 1—Lesvos (*n* = 363), 2—Samos (*n* = 51), 3—Chios (*n* = 20), 4—Ikaria (*n* = 12), 5—Fournoi (*n* = 6).

Consequently, the effect of different cultivation and oil-production parameters on the concentrations of pigments, squalene, and tocopherols were studied. As already stated, the samples originated from five islands of the Northeastern Aegean Region, Lesvos (*n* = 363), Samos (*n* = 51), Chios (*n* = 20), Ikaria (*n* = 12), and Fournoi (*n* = 6). ANOVA was performed in order to investigate whether the geographical origin was a crucial parameter to the differences presented in the concentrations of these compounds. The ANOVA showed a *p*-value < 0.05 (for a confidence level of 95%) for α-tocopherol, the sum of (β + γ)-tocopherol, β-carotene, and squalene, while it showed that the geographical origin is not a crucial parameter for lutein (*p* = 0.17). Therefore, the differentiation of the pigment and antioxidant content regarding the geographical origin is presented in Figure 2. The samples from the islands of Chios and Ikaria presented the highest average concentrations for lutein, while samples from Samos contained the highest average concentration for squalene. Regarding the tocopherols, olive oils that originated from Chios presented the highest concentrations for α-tocopherol, while samples from Lesvos contained the highest amounts for (β + γ)-tocopherols.

For the presentation of the results, box-and-whisker plots were used. In these plots, the letter x refers to the median, the thin line (whisker) marks the range of results, and the circle-spot marks the outliers.

**Figure 3:**
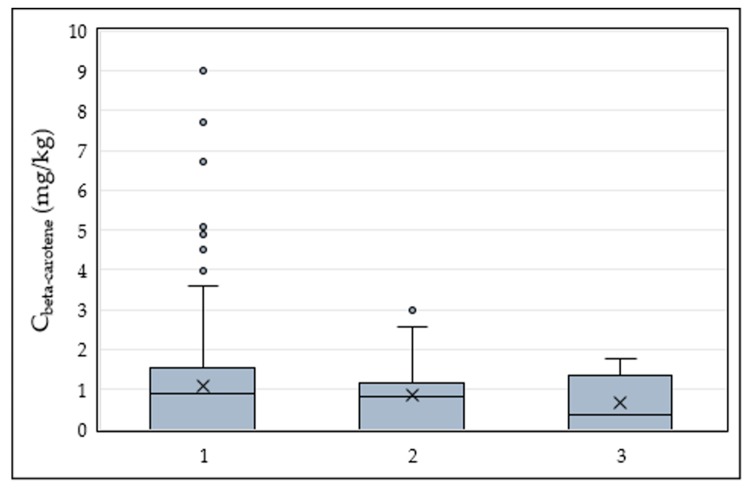
Box and whisker plot presenting the concentration of beta-carotene according to the island of origin. 1—Lesvos (*n* = 363), 2—Samos (*n* = 51), 3—Ikaria (*n* = 12).

Regarding β-carotene, it was not detected in samples that originated from Chios or Fournoi, thus they are not included in the associated boxplot (Figure 3). Samples that originated from Lesvos contained the highest amounts of beta-carotene with several samples even exceeding 5 mg/kg. The amounts of β-carotene were similar to those of olive oils produced in Italy and Chile [22,23].

**Figure 4:**
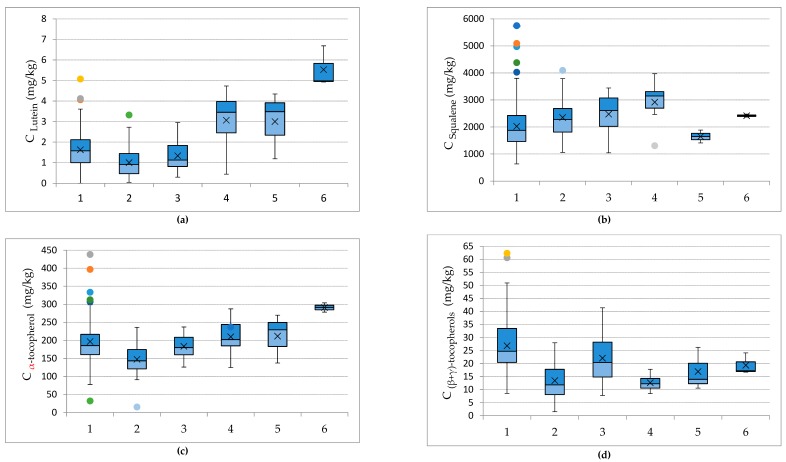
Box-and whisker plots presenting the concentrations of (**a**) lutein, (**b**) squalene, (**c**) α-tocopherol, and (**d**) (β + γ)-tocopherols in olive oil samples from different varieties. 1—Kolovi 60-100%, 2—Adramitiani 60-100%, 3—Kolovi:Adramitiani 50:50, 4—Koroneiki, 5—Ladoelia, 6- Local Wild Olive Tree.

The next parameter investigated was the variety of olive tree. Only varieties of the Lesvos island were studied as, for the other varieties, a very small number of samples were available (<3) (Table 1). For mixed oil samples containing more than one olive fruit variety, an additional study was performed to verify if there is a statistical difference compared to monovarietal samples. ANOVA was performed to compare Kolovi (100%) samples to Kolovi:Adramitiani mixtures in different ratios (60:40, 70:30, 80:20, 90:10). The results showed no significant statistical difference (*p* > 0.05) between the samples compared, and so, the samples were grouped according to the variety with the highest percentage. For Adramitiani samples, the same procedure was performed, and the results also showed no significant difference. The samples that were produced using 50:50 mixtures of Kolovi and Adramitiani were treated as a separate variety, since the ANOVA showed significant difference when compared with monovarietal samples (*p* < 0.05). Therefore, the final varieties studied were Kolovi (*n* = 239), Adramitiani (*n* = 54), Kolovi:Adramitiani 50:50 (*n* = 22), Koroneiki (*n* = 7), Local wild olive trees (*n* = 3), and Ladoelia (*n* = 3). The box-plots associated with the varieties are presented in Figure 4.

As indicated in Figure 4, the samples with the highest concentration for all the compounds belong to the local varieties, Kolovi and Local Wild Olive Tree. Kolovi has also been reported to contain increased amounts of bioactive compounds [26,27,28], indicating its exceptional nutritional value. Kolovi also presented the highest average concentrations for (β + γ)-tocopherol. The local wild olive trees and Ladoelia presented the highest average concentrations for lutein and α-tocopherol, while Koroneiki contained the highest concentrations of squalene. In comparison with monovarietal olive oils produced by Italian varieties, such as Martena, Pajarero, and Cornicabra, the average carotenoid content is twice as high [29], while the concentration levels are similar to those olive oils produced in Sicily, Italy [30]. Furthermore, the α-tocopherol content is higher in comparison with olive oils produced by olive fruits of the Spanish variety Bodocal, while it is similar to those of the Spanish varieties Negral and Racimilla [31]. For squalene, the amounts contained in olive oils of Koroneiki where similar to many varieties in the literature, while the amounts detected in Kolovi exceeded the majority of other varieties [15]

**Figure 5:**
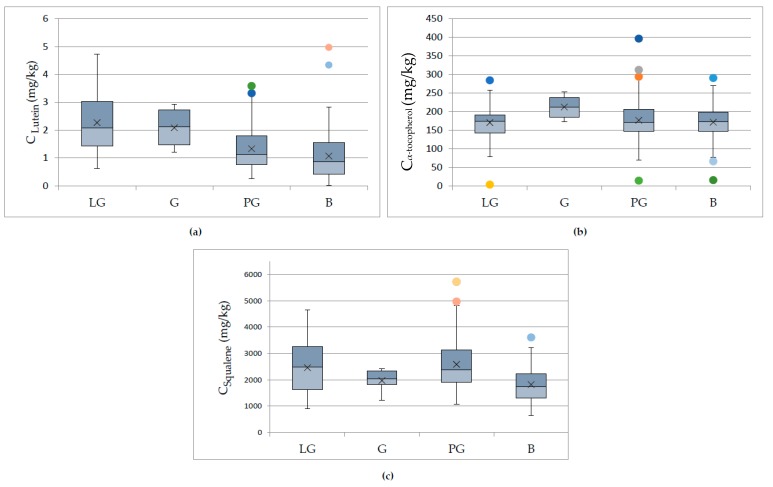
Box and whisker plots presenting the concentration of (**a**) lutein, (**b**) α-tocopherol, and (**c**) squalene in olive oils from Lesvos island regarding the maturity level of the fruit. Green (G, *n* = 4 samples), Light Green (LG, *n* = 24 samples), Purple-Green (PG, *n* = 67 samples), Black (B, *n* = 88 samples).

The next parameter that was found to affect the concentration levels of pigments and antioxidants was the maturity level of the fruit. In this study, the maturity level was assessed through the fruit color during the harvesting. The majority of the samples were produced using mixtures of fruits with different maturity levels. However, in order to obtain information about the effect of the maturity of the fruit, we compared samples that were produced using fruits of only one maturity level and originated only from the island of Lesvos. Therefore, four levels were compared: Green (G, *n* = 4 samples), light green (LG, *n* = 24 samples), purple-green (PG, *n* = 67 samples), and black (B, *n* = 88 samples). ANOVA showed that samples produced by olive fruits of different maturity level presented significant statistical differences, presenting a *p*-value < 0.05, for a confidence level of 95%, for lutein, a-tocopherol, and squalene. In Figure 5, the boxplots for lutein, α-tocopherol, and squalene content, correlated with the maturity level of the fruit, are presented. From the results obtained, it is safe to conclude that olive oil produced from olive fruits harvested at early stages of maturation presented higher pigment and antioxidant content. This conclusion confirms the results reported in various studies [11,13].

Finally, the last parameter associated with the pigment and antioxidant content in olive oils was found to be the addition of water during malaxation of the olive paste. The malaxation procedure is a very important step to the production of olive oil. As reported by Jimenez et al. [13], the purpose of this stage is to extract the oil phase from the olive paste by extracting the small drops of olive oil in order to merge and form larger drops. After the malaxation, the paste is submitted to centrifugation, in order to completely extract the olive oil. The olive oil yield and quality characteristics strongly depend on the conditions of the malaxation stage. In order to increase the oil yield, water is added to the crushed fruits, and as reported by Clodoveo [32], this can lead to a significant decrease of the concentrations of antioxidants and other bioactive compounds.

**Figure 6:**
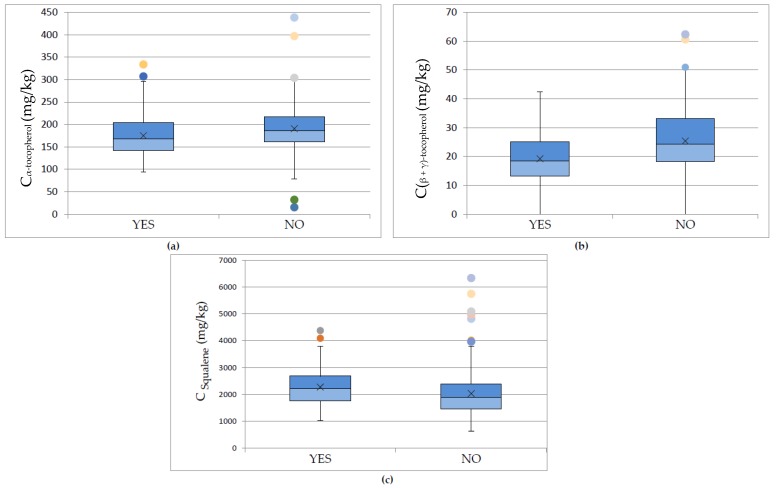
Box and whisker plots presenting the concentration of (**a**) α-tocopherol, (**b**) (β + γ)-tocopherols, and (**c**) squalene regarding to the addition of water during malaxation. YES (*n* = 120 samples), NO (*n* = 209 samples).

In this study, olive oils produced in the island of Lesvos with (*n* = 120 samples) and without (*n* = 209 samples) the addition of water were compared. The pigment compounds (chlorophylls and carotenoids) did not seem to be affected by the addition of water. ANOVA was also performed, regarding the addition of water, and the results showed a *p*-value < 0.05 (for a confidence level of 95%) for α-tocopherol, sum of (β + γ)-tocopherol, and squalene. As shown in Figure 6, the addition of water during malaxation affected the concentration of tocopherols negatively, decreasing the α-tocopherol content by about 8.5%, while the (β + γ)-tocopherols concentration decreased by about 24%. Regarding the squalene content, with the addition of water there was an increase of about 10.6%. Presumably, the addition of water leads to a better phase separation, in which the non-polar squalene is dissolved to the non-polar oily phase. On the other hand, tocopherols may not be water-soluble, but the tocopherol molecule consists of a polar head [33]. Thus, when oily and aqueous phases are in contact, tocopherols are partially distributed to the two phases, resulting in the decrease of the amounts of tocopherols in the oily phase.

As stated in the literature [24,31,32], the levels of many bioactive compounds are affected by other cultivation and oil-production parameters, such as the altitude of cultivation, biological or conventional farming, watering and/or fertilizer use, and two- or three-phase olive mill system. Also, other malaxation parameters, such as malaxation time and temperature, have been reported to be crucial for the decrease of the phenolic content and volatile compounds of olive oil [32]. However, according to the results of our study, carotenoids, tocopherols, and squalene do not appear to show a similar trend.

Overall, the results obtained from our study agree with those reported by other studies about Greek olive oils. Greek olive oils are an extraordinary source of natural antioxidants and bioactive compounds. Psomiadou et al. [34] report the monitoring of α-tocopherol, of Greek olive oils from various varieties and regions of Greece, for three consecutive harvesting years. Although in that study normal-phase-HPLC was used, the results obtained are very similar to the results we extracted. The α-tocopherol concentration of Greek olive oils ranged from 98 to 370 mg/kg, 60% of samples, >200 mg/kg. Katsoyannos et al. reported that samples of Megaritiki and Koroneiki presented very high concentrations of α-tocopherol as well. They used a reversed-phase HPLC system with a C18 column, ranging from 340 to 562 mg α-tocopherol/kg.

Regarding the pigment content, Northeastern Aegean olive oil samples present relatively higher carotenoid concentrations compared to those reported by Karabagias et al. [35], while the chlorophyll fraction results are slightly lower. A spectrophotometric method was used in that study, but the results were still comparable.

## 4. Conclusions

In this study, a novel, robust, and rapid methodology for the simultaneous determination of pigments, tocopherols, and squalene in EVOOs and VOOs was developed, optimized, and validated. The method was applied to 452 olive oil samples produced in five islands of the Greek Northeastern Aegean Region, in order to extract useful information about the concentration levels of these compounds and how they are affected by cultivation and oil-producing parameters. Overall, Northeastern Aegean EVOOs presented increased tocopherol and squalene concentrations, substantiating their exceptional nutritional value. The geographical origin proved to be a crucial parameter for the carotenoid, tocopherol, and squalene content, with Lesvos’ EVOOs and VOOs presenting relatively high concentrations for all the compounds. Specifically, samples from Lesvos presented a relatively high concentration of (β + γ)-tocopherols in comparison with the literature, while α-tocopherol content was also high. Carotenoids, tocopherols, and squalene are strongly affected by the variety of the olive fruit, with the Kolovi variety presenting the highest amounts, and the maturation level during harvesting, with olive oils produced by fruits at the early stages of maturation containing significantly higher concentrations of all compounds. The addition of water during malaxation did not affect the carotenoid fraction of olive oil; however, tocopherols and squalene were affected by this addition, resulting in the decrease of tocopherol content and in the increase of squalene concentration. A relatively low chlorophyll concentration was present in all samples, which was not affected by cultivation or oil-production parameters. Last but not least, other parameters of cultivation and oil-production that have been investigated during this study, such as altitude of cultivation, biological or conventional farming, watering and/or addition of fertilizer, and two- or three-phase centrifugation, did not have any impact on the concentration levels of pigments, tocopherols, and squalene.

## Figures and Tables

**Table 1:** Retention times and wavelength of absorption of carotenoids, chlorophylls, tocopherols, and squalene.

Compound	Wavelength (nm)	Retention Time (min)
Carotenoids	430 and 450	Lutein: 6.5 β-carotene: 22.5
Tocopherols	295	α-tocopherol: 11.0 γ-tocopherol: 10.0 δ-tocopherol: 9.0
Chlorophylls	410 and 660	Chlorophyll a: 10.0 Chlorophyll a’: 11.0 Pheophytin a: 18.0 Pyropheophytin: 25.0
Squalene	210	Squalene: 16.5

**Table 2:** Calibration curves and correlation factors for each analyte.

Compound	Concentration Range (mg/L)	Calibration Curve	Correlation Factor (r)
β-carotene	0.1–5.5	y = 111.6 (±3.1) x +11.8 (± 8.6)	0.998
lutein	0.01–10	y = 198.70 (±0.84) x +7.4 (±3.9)	0.999
α-tocopherol	2–140	y = 7.373 (±0.049) x −1.3 (±3.3)	0.9998
γ-tocopherol	0.5–20	y = 9.382 (±0.030) x −0.27 (±0.26)	0.99994
δ-tocopherol	1–100	y = 8.14 (±0.21) x −7.7 (±10.9)	0.998
squalene	20–600	y = 66.5 (±2.6) x +244 (±301)	0.997
chlorophyll	0.5–10	y = 42.72 (±0.68) x +3.7 (±3.5)	0.9995

**Table 3:** %Recoveries (%R) for evaluation of repeatability.

Compound	Low Concentration (%R ± SD ^1^) (*n* = 6)	Medium Concentration (%R ± SD ^1^) (*n* = 6)	High Concentration (%R ± SD ^1^) (*n* = 6)
β-carotene	94.3 ± 4.9	96.6 ± 9.4	82.1 ± 1.8
lutein	96.6 ± 1.0	91.1 ± 3.6	88.6 ± 3.9
α-tocopherol	98.2 ± 6.6	95.1 ± 6.4	96.7 ± 2.6
γ-tocopherol	98.6 ± 4.2	95.7 ± 6.1	93.2 ± 1.5
δ-tocopherol	105.9 ± 4.0	107.6 ± 1.8	106.9 ± 2.8
squalene	109.3 ± 3.8	101.0 ± 6.4	92.0 ± 5.6
chlorophyll	90.4 ± 5.1	91.8 ± 1.4	89.6 ± 1.5

^1^ SD: Standard Deviation.

**Table 4:** %Recoveries (%R) for evaluation of intermediate precision.

Compound	Low Concentration (%R ± SD ^1^) (*n* = 18)	Medium Concentration (%R ± SD ^1^) (*n* = 18)	High Concentration (%R ± SD ^1^) (*n* = 18)
β-carotene	104.3 ± 10.2	97.2 ± 10.8	100.1 ± 15.3
lutein	93.5 ± 4.6	92.7 ± 3.2	91.0 ± 4.3
squalene	102.7 ± 7.5	102.1 ± 8.4	104.2 ± 7.2
α-tocopherol	99.5 ± 3.6	100.0 ± 6.0	99.6 ± 6.4
γ-tocopherol	105.3 ± 4.6	105.6 ± 5.2	104.2 ± 6.6
δ-tocopherol	103.6 ± 6.2	99.2 ± 4.4	96.0 ± 5.0
chlorophyll	101.1 ± 10.3	100.4 ± 7.9	102.1 ± 10.3

^1^ SD: Standard Deviation.

**Table 5:** Limit of detection (LOD) and limit of quantitation (LOQ) of each compound.

Compound	LOD ^1^ (mg/L) (*n* = 10)	LOD (mg/kg) (*n* = 10)	LOQ ^2^ (mg/L) (*n* = 10)	LOQ (mg/kg) (*n* = 10)
β-carotene	0.030	0.280	0.090	0.830
lutein	0.005	0.050	0.013	0.140
squalene	3.70	35.1	11.1	105.3
α-tocopherol	0.65	5.30	1.70	15.8
γ-tocopherol	0.1	3.50	0.4	10.6
δ-tocopherol	0.200	1.20	0.600	3.70
chlorophyll	0.150	1.30	0.450	3.90

^1^ LOD: limit of detection, ^2^ LOQ: limit of quantitation.

**Table 6:** Pigment, tocopherols, and squalene results.

Compound	Number of Samples ^1^	Concentration Range (mg/kg)	Average Concentration (mg/kg)	SD ^4^ (mg/kg)	Median (mg/kg)
β-carotene	290	0.09–9.00	1.53	1.07	1.18
Lutein	452	LOQ ^3^ < −7.43	1.59	0.95	1.50
Squalene	452	635–6344	2195	831	2109
α-tocopherol	452	LOQ < −438	180	48	179
(β + γ)-tocopherol ^2^	410	LOQ < −62	22	11	21

^1^ samples in which the compounds were detected; ^2^ (β + γ)-tocopherols co-elute; thus, they are quantified as a sum. ^3^ LOQ: limit of quantitation, ^4^ SD: Standard Deviation

## Supplementary Materials

The following are available online at https://www.mdpi.com/2304-8158/9/1/31/s1, Figure S1: Sampling Zones of the island of Lesvos, Figure S2: Pheophytin and Pyropheophytin Spectra acquired (**a**) from Hornero- Mendez et. al. [18] and (**b**) our HPLC-DAD system, Table S1: Categorization of Samples regarding cultivation and olive-oil production parameters (Full), Table S2: Peak Symmetry using the gradient elution program, for lutein, chlorophyll and beta-carotene.
