# Supplementary material for: Simultaneous Determination of Pigments, Tocopherols, and Squalene in Greek Olive Oils: A Study of the Influence of Cultivation and Oil-Production Parameters
Source: Foods. 2019 Dec 29;9(1):31. doi: 10.3390/foods9010031 (PMC7023276; doi:10.3390/foods9010031)
Supplement: Supplementary file 1 [file foods-09-00031-s001.pdf]

Simultaneous determination of pigments, tocopherols and squalene in Greek olive oils: A study of the influence of cultivation and oil-production parameters

Ioannis C. Martakos, Marios G. Kostakis, Marilena E. Dasenaki*, Michalis I. Pentogennis and Nikolaos S. Thomaidis

Electronic Supplementary Material

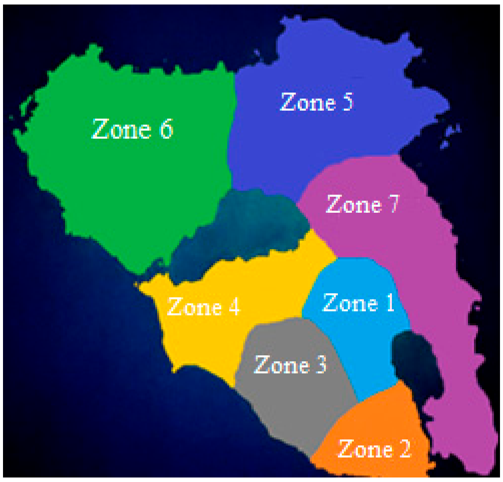

Figure S1. Sampling Zones of the island of Lesvos

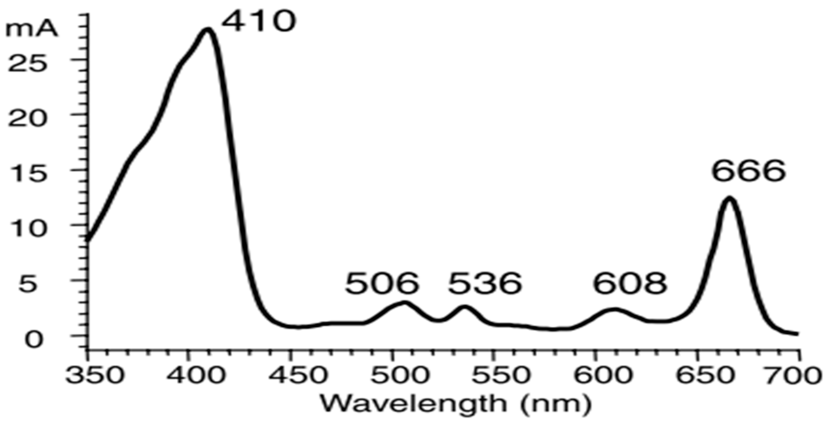

(a)

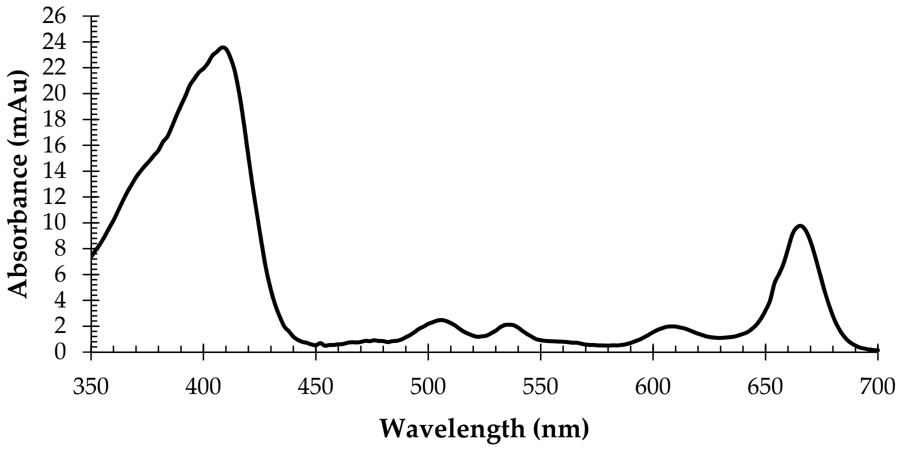

(b)

Figure S2. Pheophytin and Pyropheophytin Spectra acquired (a) from Hornero- Mendez et. al. [18] and (b) our HPLC-DAD system

Table S1. Categorization of Samples regarding cultivation and olive-oil production parameters **(Full)**

	Categories	Number of Samples
**Geographical Origin**	Lesvos	363
	Samos	51
	Chios	20
	Ikaria	12
	Fournoi	6
**Sampling Zones (Lesvos Island)**	1	30
	2	112
	3	59
	4	20
	5	14
	6	49
	7	64
**Olive tree variety**	Local Wild Olive tree	3
	Adramitiani	36
	Arbequina	1
	Dafnoelia	2
	Throumpa	17
	Kolovi	169
	Koroneiki	45
	Ladoelia	3
	Leccino	1
	Manaki	1
	Patrino	1
	Chiotiki	4
	Chondrolia	1
**Altitude of Cultivation**	High	119
	Medium	126
	Low	90
**Cultivation**	Conventional	292
	Biological	130
**Cultivation Treatment**	Water	71
	Fertility	101
	None	229
**Maturity stage during ripeping**	Light-Green	34
	Green-Blue	67
	Black	88
**Days from harvesting to oil production**	0	59
	1	65
	2-3	183
	4-6	57
	7-10	16
**Addition of water during malaxation**	Yes	154
	No	243
**Malaxation Time (min)**	20-35	72
	40-55	157
	60-75	143
	80-100	32
**Malaxation Temperature (°C)**	20-29	149
	30	145
	31-40	110

**Type of Centrifugion**	2-phase	162
	3-phase	278
**Decanter Temperature (^{o}C)**	15-29	81
	30	81
	31-45	214

Table S2. Peak Symmetry using the gradient elution program, for lutein, chlorophyll and beta-carotene

Mobile Phase	Compound	Retention Time (min)	Peak Symmetry
Gradient	Lutein	6.0	1.12
	Chlorophyll a	10.0	1.18
	Beta carotene	22.5	0.84
Isocratic elusion	Lutein	6.0	1.1
	Chlorophyll a	10	0.79
	Beta carotene	27.5	0.66
